# Unraveling the Phosphorus Adsorption Mechanisms in Three-dimensional Reduced Graphene Oxide Materials

**DOI:** 10.1021/acs.langmuir.4c00810

**Published:** 2024-05-16

**Authors:** Patrick R. B. Côrtes, Mayara Bitencourt Leão, Gabriel Lopes Rezende Reis, Douglas D. de Vargas, Gabriel Fidencio Murillo, Mateus H. Köhler, Carolina Ferreira de Matos Jauris

**Affiliations:** † Department of Physics, Federal University of Santa Maria, Santa Maria 97105-900, Brazil; ‡ Environmental Science and Technology Center, Federal University of Pampa, Caçapava do Sul 96570000, Brazil; § Department of Chemistry, Federal University of Santa Maria, Santa Maria 97105-900, Brazil

## Abstract

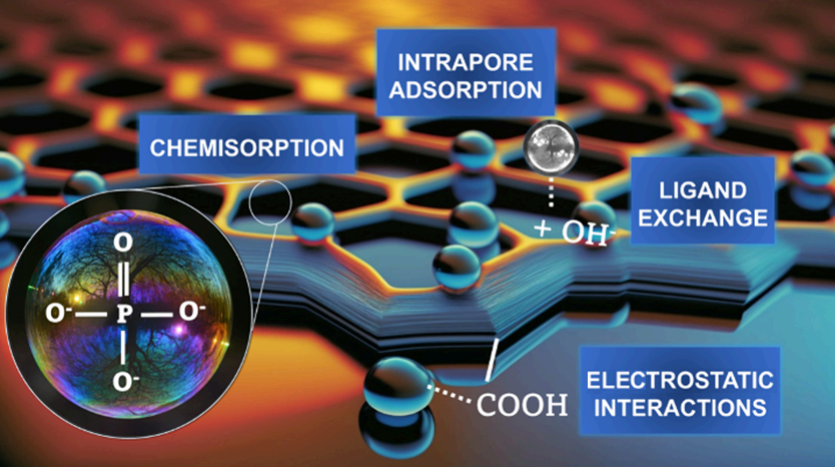

To prevent eutrophication, controlling the phosphate concentration levels is one of the most important issues in surface water management. One of the most utilized methods is phosphate adsorption. However, its application faces a bottleneck due to the unclear understanding of adsorption and interaction mechanisms. The present work unlocks the phosphorus adsorption mechanisms in three-dimensional reduced graphene oxide with different reduction levels and pore sizes to remove phosphate from water using experiments and multiscale simulations. Experiments were performed to evaluate the influence of pH, ionic strength, and temperature on the adsorption. Molecular Dynamics and Ab Initio simulations evaluated the influence of the pore size and oxidation degrees of the materials. We show that the adsorption capacity of the materials increases with increasing pH and ionic strength and decreasing temperature. It is observed that the more oxidized the material and the less compact the structure, the better the adsorption. These results are theoretically explained in terms of the interaction of functional groups and the clustering of phosphate ions, which results in better adsorption in materials with larger pores. The underlying mechanisms for the 3D-reduced graphene oxide performance were confirmed by spectroscopy analysis. All the results show that 3D-reduced graphene oxide can sorb phosphate in different complex water remediation systems with characteristics that can be modulated by changing the material synthesis method.

## Introduction

Phosphorus (P) is essential for life, and its cycle is one of the most relevant biogeochemical processes affecting all ecosystems.^1^ However, anthropogenic activities associated with rapid population growth and industrial development have significantly altered the global phosphorus cycle. Large amounts of phosphorus, mainly in phosphate ion form (PO_4_^–3^_(aq)_), are released during human activities such as phosphate mining, fertilizer production, farming-related processes, food manufacturing, and waste treatment.^2,3^ The interaction between phosphorus and several ecosystems worldwide has negatively impacted available water quality.^1,4^ At the same time, there is an increasing need for water suitable for human consumption.^4^ The phosphate ion is a limiting factor for water eutrophication. The excess of PO_4_^–3^_(aq)_ stimulates the overproduction of algae and phytoplankton, impacting water quality, reducing dissolved oxygen, and leading to the death of aquatic organisms.^3,5−9^ All this potentially harms the water balance in lakes and oceans worldwide.^10^ Even the proliferation of cyanobacteria and harmful algal blooms can be triggered in more severe cases.^9^

Several methods to remove phosphate from water have been investigated in recent decades. The methods mainly include biological, phytoremediation, precipitation, and adsorption.^5,10^ Among them, adsorption presents a low cost associated with high flexibility, design simplicity, and selectivity, making it ideal for sustainable phosphorus removal from effluents.^5,11,12^ Unlike other techniques, it is also an excellent treatment for water contaminated with low phosphate levels^12^ since environmentally relevant phosphate concentrations range from trace levels to a few milligrams per liter. Bacelo et al.^12^ reviewed many materials such as metal oxides/hydroxides, carbonate minerals, clay minerals, zeolites, porous silica, activated carbon, biochar, polymers, bioderived materials, and industrial waste that have been used in the adsorption of phosphate. More recently, graphene-based materials functionalized with different compounds have been successfully applied to phosphate adsorption from water. Highlighting studies carried out with composite materials, reduced graphene oxide doped with N and decorated with Fe_3_O_4_, zirconium-loaded reduced graphene oxide, triazine functionalized graphene oxide anchored alginate bead, cellulose acetate nanofiber membrane modified with graphene oxide/sodium dodecyl sulfate, alumina decorated graphene oxide, lanthanum-doped aminated graphene oxide@aminated chitosan microspheres, among others, showing excellent performance for all tested materials.^13−22^ However, despite the excellent performance obtained by two-dimensional (2D) graphene materials and their extraordinary surface-to-volume ratio, two main problems hinder their application: (i) van der Waals interactions between graphene sheets cause their aggregation,^23^ which reduces the superficial area and (ii) for applications in aqueous systems, graphene sheets can be carried away, leading to material loss and possible ecotoxicological damage. One alternative is using three-dimensional (3D) materials based on graphene.

3D graphene is formed by the disorderly bonding of graphene sheets, forming a porous network with interconnected pores, high surface area, and stability in aqueous media. Therefore, they are promising materials for removing different contaminants from water.^24^ Recently, Zhang et al.^25^ evaluated phosphate removal with three-dimensional graphene. They used a hybrid cellulose/graphene nanomaterial functionalized with bimetallic hydroxides for pH interference and competitive adsorption with various anions and organic matter. However, the adsorption mechanism is yet to be clarified. The absence of studies presenting phosphorus adsorption mechanisms in graphene-based structures is remarkable.

In this regard, computational studies have provided a fundamental microscopic perspective into adsorption processes at the solid–liquid interface. Several molecular dynamics (MD) and first-principles DFT-based simulations have been employed to elucidate adsorption phenomena in nanomaterials with tremendous success.^26−28^ The open questions regarding the phosphate adsorption mechanisms in graphene-based nanomembranes represent a considerable opportunity for MD and hybrid Ab Initio MD (AIMD) simulations that will be further explored in this contribution.

In this work, we used tridimensional reduced graphene oxide materials with different oxidation degrees to remove phosphorus from water, evaluating the influence of temperature, pH, and ionic strength on the adsorption efficiency. In addition, classical and quantum-based simulations were employed to elucidate the adsorption mechanism from a microscopic point of view. Finally, the materials were applied to remove phosphorus in a natural lake eutrophicated sample, obtaining good results and confirming the applicability of the materials in a natural environment.

## Materials and Methods

### Experimental Section

#### Obtaining Tridimensional Reduced Graphene Oxide Materials

The four different 3D materials used in this work were obtained as described in our previously published works.^29,30^ The synthesis started from the oxidation of graphite (<20 μm, purchased from Sigma-Aldrich), followed by exfoliation of the graphite oxide through an ultrasound bath to obtain graphene oxide (GO). Then, 50 mL of a GO dispersion with approximately 1 mg mL^–1^ was placed in beakers using different concentrations of ascorbic acid as the reducing agent (0, 5, 10, and 25 mmol L^–1^). These beakers were placed in an autoclave for 90 min at 120 °C. After about 2 h, the materials with the three-dimensional structure already formed were removed from the autoclave and washed with distilled water until they were at neutral pH. Finally, the materials were unrestrictedly dried in an oven at 60 °C for 24 h.

#### Materials Characterization

Three-dimensional materials were characterized through Scanning Electron Microscopy (SEM) using a MIRA 3 FEG-SEM microscope. The samples were placed on double-sided copper tape, previously glued to the sample holder. All samples were metalized with chromium; the source voltage was 15 kV.

The 3D-reduced graphene oxide materials before and after the adsorption and the KH_2_PO_4_ salt were analyzed on a Vertex-70 (Bruker) in attenuated total reflectance (ATR) mode using an ATR accessory (Pike Technologies). The Raman spectra were obtained on a SENTERRA confocal Raman microscope (Bruker) equipment, with the laser emitting at 633 nm. The adsorption process for the spectroscopic studies was performed at 22 °C, pH = 7, and without salt for ionic strength adjustment.

The degree of functionalization was estimated through indirect potentiometric titration and by thermogravimetric analysis, which quantifies acidic functional groups (−COOH). The materials were left in a 0.0025 mol L^–1^ NaOH solution, standardized with potassium biphthalate, for 24 h. Afterward, the solution was filtered and titrated with HCl 0.001 mol L^–1^, using KCl 0.01 mol L^–1^ as a supporting electrolyte. The pH of the zero-charge point was determined using a 0.05 mol L^–1^ NaCl solution with pH values between 1 and 9. The materials were kept in solution for 48 h, and the pH variation was calculated. Thermogravimetric analyses (TGA) were carried out on a Netzsch TG 209 F1 equipment under a nitrogen atmosphere, starting from room temperature to 900 °C, at a heating rate of 10 °C min^–1^.

### Adsorption Study

The influences of pH, ionic strength, and temperature on adsorption were evaluated. Ten mg of each material and 5 mL of 100 μmol L^–1^ K_2_HPO_4_ solution were used for each test. After the mixture, the system was kept under stirring for 24 h. The remaining concentration of K_2_HPO_4_ in the solution was verified at the end of the contact time. Quantification was performed using a Kasuaki IL-593 UV–vis spectrophotometer and the methodology described by Rice et al.^31^ The calibration curve is presented in the Supporting Information (Figure S1).

To evaluate the pH effect on adsorption, 100 μmol L^–1^ K_2_HPO_4_ solutions were adjusted to pH 3, 7, and 12 using HCl 0.05 mol L^–1^ or NaOH 0.01 mol L^–1^. Furthermore, the effect of ionic strength was evaluated by comparing adsorption in samples with NaCl concentrations of 0, 0.1, and 1.0 mol L^–1^. Finally, the temperature effect was evaluated by keeping the samples at 4 °C, 22 °C, and 50 °C.

Adsorption tests evaluated the stability of the adsorptive process carried out during 24 h, followed by desorption tests for another 24 h, at pH = 7, temperature of 22 °C without adding NaCl.

The adsorptive capacities were calculated according to eq 1, where *Q_e_* is the adsorptive capacity (mg g^–1^), *C_e_* is the initial concentration (mg L^–1^), and, *C_f_* is the final concentration (mg L^–1^), *V* is the volume (mL), and *m* is the mass of material (in grams):
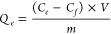


### Application in an Environmental Sample

A sample was collected in a eutrophicated lake at the Federal University of Pampa, Caçapava do Sul, RS, Brazil (30°29′49.1″S 53°28′54.8″ W). Approximately 1 L of water was obtained in an amber glass bottle previously washed thoroughly and left in a 10% nitric acid bath for 10 h. The bottle was acclimatized 3 times to obtain the water by rinsing and discarding the sample downstream of the site. First, the concentration of phosphorus in the solution was quantified. Then, ion adsorption studies were carried out on the material. 5 mL of the real sample was placed in test tubes with 10 mg of each material. After 24 h of stirring, the phosphorus concentration was quantified, and the adsorption efficiency was defined.

### Simulation Details

**Figure 1:**
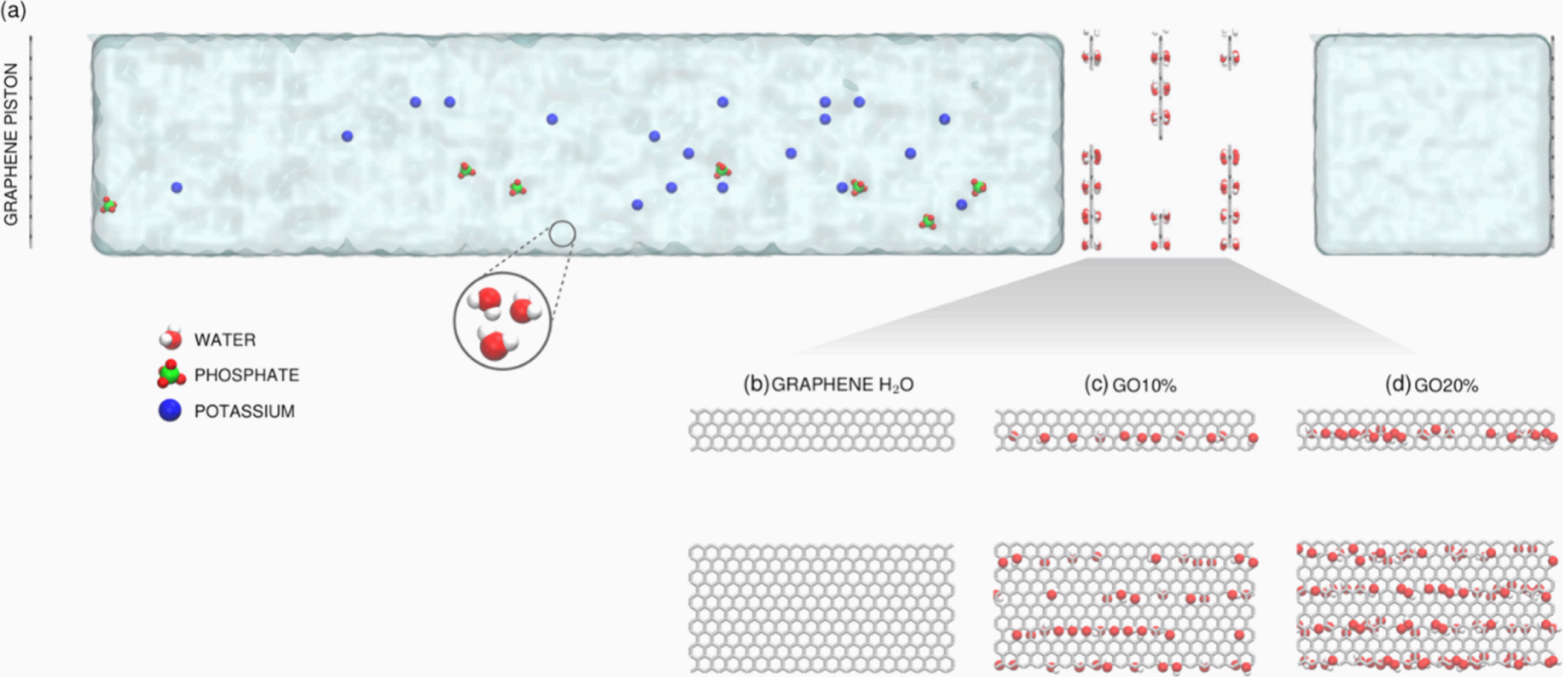
(a) The initial computational setup for the pressure-driven water flux through (b) graphene, (c) GO10% and (d) GO20%.

MD simulations were performed using the Large-scale Atomic/Molecular Massively Parallel Simulator (LAMMPS).^32,33^Figure 1 shows the simulation setup with the membrane between two water reservoirs. To ensure that water molecules fill in the membrane, we employ graphene sheets as pistons to control the confined solution pressure (similar to previous works^33,34^).

The materials obtained were named according to the reducing agent concentration used in the synthesis in the experimental part. The 3D-rGO0 material, for example, does not contain ascorbic acid; 3D-rGO5 contains 5 mmol L^–1^ of ascorbic acid, 3D-rGO10 contains 10 mmol L^–1,^ and 3D-rGO25 contains 25 mmol L^–1^.^29^ The reader can check the relation between the concentration of the reducing agents and the number of oxygenated groups in Table S1 of the Supporting Information.

To compare and assess different oxidation regimes in the MD simulations, we investigated three membrane architectures: Graphene, GO10%, and GO20%. Here, the GO10% and GO20% systems are pristine graphene sheets where 10 or 20% of their carbon atoms, respectively, are connected to either a hydroxyl or an epoxy group, as seen in Figure 1c,d. The simulations with the GO20% membrane represent the experimental 3D-rGO0 (where no reduced agents are used and approximately 26% of oxygenated groups remain). In contrast, the simulations with pristine graphene membranes are associated with the 3D-rGO25 due to the high degree of reducing agents. Furthermore, during the simulations, the interlayer spacing, defined as the C–C distance between adjacent graphene or GO layers, is set to 1.5 nm for Graphene and GO20% membranes and varies between 1.5 and 2.0 nm for GO10% membranes. Considering the center-to-center edge carbon distances, the investigated nanopores are 1.5 and 2.0 nm wide, respectively.

The initial system (from piston to piston) has 5 × 5 × 33 nm in *x*, *y*, and *z* axes, respectively, and contains 9000 water molecules, with 7000 in the feed reservoir. Water interactions were modeled by SPC/E. LJ interaction parameters for all the atomic species are summarized in Table 1. Lorentz–Berthelot mixing rules were employed for the nonbonded interactions. The long-range electrostatic interactions were calculated by the particle–particle–particle–mesh method, and the LJ cutoff distance was set to 1.2 nm. The SHAKE algorithm maintained water and phosphate molecules rigid with a time step of 0.5 fs.

**Table 1:** LJ Parameters and Atomic Charges Are Employed in This Work

	σLJ (Å)	ε (kcal/mol)	charge (e)
C^35^	3.40	0.086	0
O^36^	3.166	0.1553	–0.8476
H^36^	0	0	+0.4238
P^37^	3.8435	0.08583	0
O (phosphate)^36^	3.166	0.1553	–0.75
O (epoxi)^38^	3.166	0.1553	–0.36
O (hydroxyl)^38^	3.166	0.1553	–0.57
H (hydroxyl)^38^	0	0	+0.18
K^39^	2.86	0.23018	+1

The MD simulations were carried out as follows. First, each system was energy minimized for 0.5 ns at a constant number of particles, volume, and temperature (NVT) ensemble. Then, each reservoir was equilibrated in the NPT ensemble for two ns at 1 bar and 300 K. The external pressure was simulated by leaving the pistons free to move only in the *z*-direction under applying a force in the same direction to produce the desired pressure. At this stage, water fills the membrane, and each reservoir reaches the equilibrium density of approximately 1 g cm^–3^. After that, the pistons are held fixed in space again, and the systems are equilibrated in the NVT ensemble for 2 ns at 300 K. The Nosè-Hoover thermostat was used with a time constant of 0.1 ps. Next, nonequilibrium simulations were implemented to calculate the water pressure-driven flow across the membranes. The pistons create a 1000 bar gradient pressure along the simulation box for 10 ns. During this step, both the density profiles and adsorption rates are obtained. Each calculation was averaged over three sets of simulations with different initial thermal velocity distributions.

We also performed Ab Initio Molecular Dynamics (AIMD) simulations to check the systems’ thermal stability. The first-principles calculations were implemented in the QUANTUM ESPRESSO package.^40,41^ Interactions between the valence electrons and the atomic core were modulated by the projector augmented wave (PAW) method.^42^ The exchange-correlation functional ε_XC_ was calculated through the generalized gradient approximation (GGA) in the Perdew-Burke-Ernezhof (PBE) form.^43^ The long-range van der Waals (vdW) interactions were described by the vdW-DF2 functional^44^ with c09 exchange correction.^45^ We used a vacuum region of 2 nm in the three directions to avoid interactions between periodic images. Twenty-five ps of NVT-AIMD simulations at 300 K were performed with a time step of 1 fs.

## Results

### The Physical Aspect and Material Characterization

**Figure 2:**
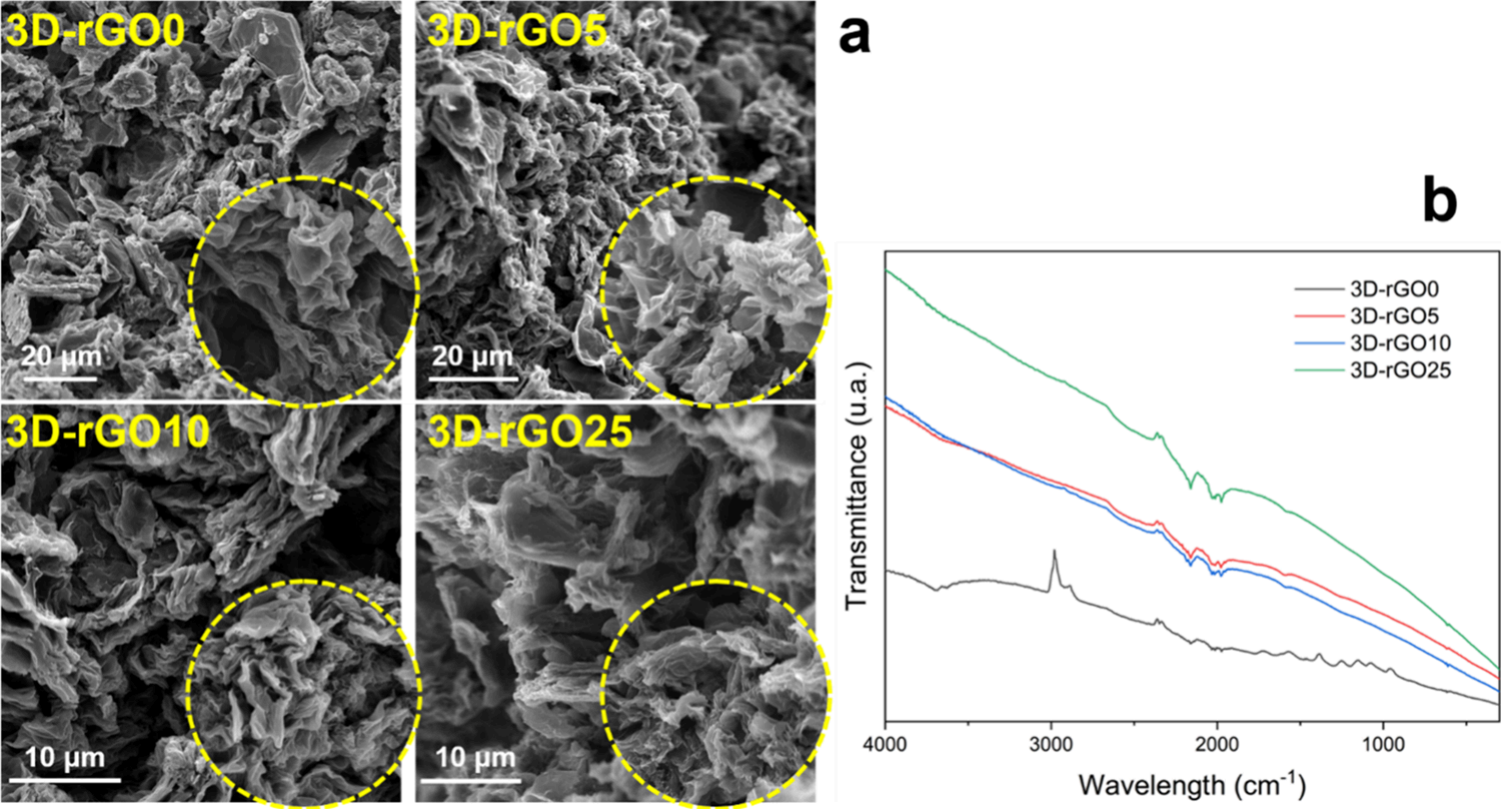
Scanning electron microscopy images of samples 3D-rGO0, 3D-rGO5, 3D-rGO10, and 3D-rGO25 (a) and FTIR spectroscopy of these materials (b).

As shown in previous work,^29^ the synthesis process made it possible to obtain three-dimensional graphene-based materials in a simple, fast way, with the need for only one step at a low cost, without using toxic reagents and templates. In addition, the material obtained presented a cohesive and self-supporting structure. From the scanning electron microscopy images, it was possible to observe the microscopic structure of the materials. Figure 2a shows the SEM images for materials 3D-rGO0, 5, 10, and 25. Due to the pressure and temperature provided during the synthesis, we observed a three-dimensional structure formed by the disordered bonding of the reduced GO sheets. It is evident from the analysis of the figures that the greater the amount of reducing agent added during the synthesis, the more compact the structure and the smaller the pores. Hence, the pore sizes for materials with reducing agents are 3D-rGO5 > 3D-rGO10 > 3D-rGO25. For the 3D-rGO0 material, we observed no cohesive structure formation due to the absence of a reducing agent during the synthesis. Instead, a granular material that resembles crumpled graphene is obtained, as shown and detailed by Leão et al.^29^ The specific surface area data obtained by BET corroborate these data and show that the more compact the structure by SEM, the smaller the specific surface area, with values ranging from 62.8 to 172.3 m^2^ g^–1^ (Table 2).^29^ The formation of the three-dimensional structure, with the sheets arranged in a disordered manner, is proven by X-ray diffraction (XRD). The diffractograms, shown in Figure S2, show a wide variation in interplanar distances that represent the organization of the sheets, without restacking, during the thermochemical reduction process.

**Table 2:** Properties of the Materials Obtained According to the Characterizations Performed^29^

material	concentration of COOH (mmol g^–1^)	pH_ZCP_	specific surface area (m^2^ g^–1^)
3D-rGO0	10.62 ± 1.84	2.00	172.274
3D-rGO5	3.08 ± 0.12	2.10	163.198
3D-rGO10	2.89 ± 0.11	2.22	68.980
3D-rGO25	2.52 ± 1.34	2.37	62.807

The four infrared spectra (presented in Figure 2 b and further discussed in Figure 8) showed mainly bands associated with characteristic oxygenated graphene functional groups.^29^ In addition, we observed a decrease in the intensity of these bands with an increase in the reducing agent. The results are supported by thermogravimetric analysis (Figure S2), where it can be observed that the higher the concentration of the reducing agent used, the lower the total amount of oxygenated groups (Table S1): 26.1, 11.4, 8.5, and 5.1 wt % for the 3D-rGO0, 3D-rGO5, 3D-rGO10 and 3D-rGO25, respectively.

Corroborating TGA data, the potentiometric titration (Table 2) showed that the greater the amount of reducing agent added, the more efficient the reduction process. The 3D-rGO0 material has more functional groups because it uses only thermal synthesis. These results also ratify those of pH_ZCP_, as the more oxidized the structure, the lower the pH_ZCP_ (Table 2), with values ranging between 2.00 and 2.37. These characterizations allow a better understanding of the results, which will be discussed later.

### Experimental Adsorption

First, it is important to point out that this study aims to compare the effect of different ionic strengths, temperatures, and pH on the adsorption of materials with different morphologies and degrees of oxidation. It is not an objective to contemplate the realization of conventional studies, such as adsorption time and maximum adsorptive capacity. This explains why the adsorptive capacities, although in the same order of magnitude as those described in the literature, are low. Also, this low adsorption is believed to be related to the drying method of the three-dimensional structure. This study used the materials as xerogels, with higher compaction and lower adsorptive capacities than those in a hydrogel form used in our previous studies.^29,46−48^

**Figure 3:**
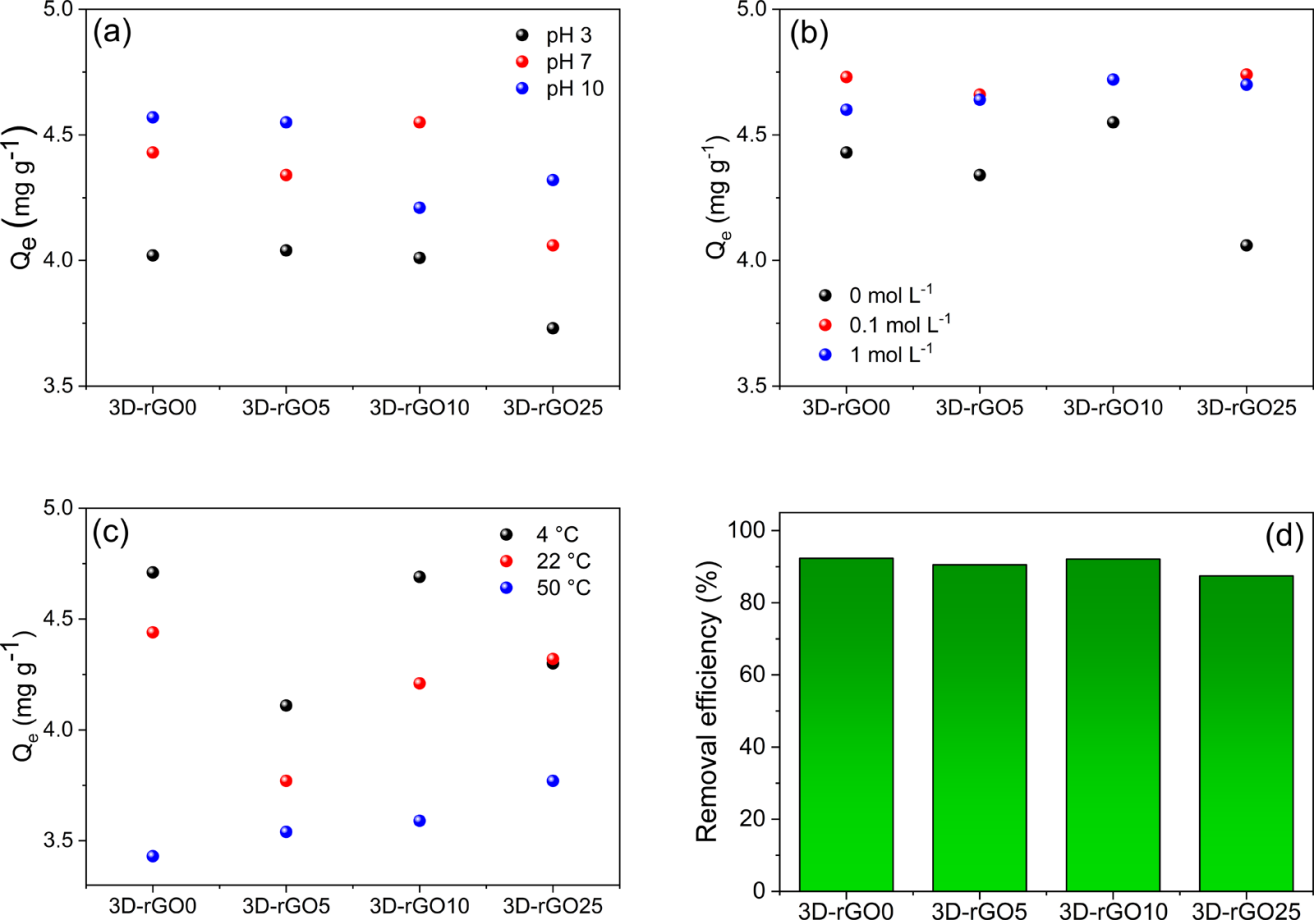
Comparison of adsorptive capacities from the influence of (a) pH, (b) ionic strength, and (c) temperature variations. In (d), the phosphate removal efficiency from an environmental sample. Conditions: 10 mg of material with 5 mL of 100 μmol L^–1^ K_2_HPO_4_, constant stirring for 24 h.

In Figure 3, the experimental results for the adsorption of the phosphate ion are presented. The pH interference was evaluated in acidic (pH 3), neutral (pH 7), and basic (pH 10) solutions, and the results are shown in Figure 3a. Lower removal efficiency is generally observed when the medium is acidic. The highest removal efficiencies are observed in basic media, except for the 3D-rGO10 material, which presents the best results in neutral media. Therefore, anionic contaminants are proposed to be adsorbed onto adsorbents through specific and/or nonspecific adsorption. Specific adsorption involves ligand exchange reactions, where anions displace OH groups from the surface.^49^ In specific adsorption, also called ligand exchange or chemisorption, a strong covalent bond is formed due to the ion binding directly to the surface without a water molecule interposed between them. Thus, adsorption occurs on positive, negative, or neutral surfaces.^50^ In the case of nonspecific adsorption or electrostatic attraction, the main dependence is on the pH of the adsorbent. A previous study where the adsorption of phosphate ion in graphene was evaluated also obtained better adsorption results at pH > pH_ZCP_, indicating that adsorption occurs even if the surface is electrically negative.^49^ It is, therefore, suggested that the adsorption of phosphate by the evaluated materials is mainly a specific process.

The interference of ionic strength was also evaluated (Figure 3b), varying the NaCl concentrations in 0.1 and 1 mol L^–1^, in addition to comparing the results with an experiment without the addition of NaCl (0 mol L^–1^). In these experiments, we observed that the lowest removal efficiencies are found when NaCl is not added. However, regardless of the concentration added, the values are very similar when salt is added, with greater differences observed for 3D-rGO0. Such results indicate a greater efficiency of phosphate ion removal in brackish waters and marine environments. Still, the removal efficiency is relatively close for 3D-rGO0, 3D-rGO5, and 3D-rGO10 materials, with or without NaCl addition. A similar behavior was observed in a previous study, where phosphate adsorption increased by increasing the ionic strength.^50^

Similarly, the degree to which the ionic strength influenced the adsorption of the two solutes was not significant. The higher adsorption at higher NaCl concentration was attributed to the increase in the electrical potential at the interface, induced by the surroundings of Na^+^ cations, since the increase in the electrical potential increases the electrostatic attraction toward the negatively charged phosphate, favoring the adsorption process.^51^ In addition, phosphate solubility is lower at higher ionic strength due to the salting-out effect, which could at least partially contribute to the increased adsorption^52,53^ by the greater phosphate availability in the solution.

Finally, the effects of temperature variation on phosphate ion adsorption were evaluated, and the results are shown in Figure 3c. It is evident that temperature significantly influences phosphate adsorption in materials based on 3D graphene. The lower the temperature during adsorption, the more efficiently the materials remove phosphate. Previous studies evaluated the thermodynamic parameters of phosphate adsorption, and it was found that the efficiency of phosphate adsorption decreases with increasing temperature.^54,55^ This demonstrates higher efficiencies at low temperatures.^56,57^

When comparing the materials, it is identified that 3D-rGO25 is the material that has the lowest adsorptive capacity compared to the others, except when NaCl is added. In studying the influence of pH, all three other materials behave similarly. For the temperature study, the 3D-rGO5 material presents the lowest removal efficiencies, but generally, this material has a smaller variation in the results. This indicates that this material can be less influenced by the parameters tested, with a greater influence of other parameters, as discussed later. For all results, there is a tendency for a better performance of the 3D-rGO0 material, which can be explained in terms of the greater presence of oxygenated functional groups. They assist in the adsorption process with a more significant interaction with the phosphate ion compared to the graphene structure.

When evaluating the percentage of phosphate desorption (Figure S4), it was observed 7.4, 11.3, 2.1, and 4.6% desorptions of the total adsorbed phosphate for 3D-rGO0, 3D-rGO5, 3D-rGO10, and 3D-rGO25 materials, respectively. This indicates a strong interaction and stability of phosphate with all materials.

The applicability of these materials was performed in a real lake water sample; the total phosphate concentration determined for this sample was 2.6 mg L^–1^, consistent with a eutrophic environment. The phosphate removal from this sample exceeded 87% in all materials (Figure 3d). Overall, the 3D-rGO25 material showed the lowest removal efficiency, followed by the 3D-rGO5 material. The most efficient material tested was 3D-rGO0 (92.3%), the most oxidized material. These results indicate that the materials can be successfully applied to real samples and may allow the treatment of effluents with phosphate ions.

### Clusterization as the Driving Mechanism

**Figure 4:**
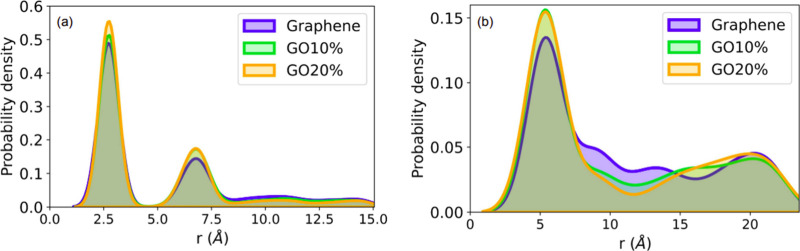
(a) P–K and (b) P–P atomic radial distribution function from MD simulations.

**Figure 5:**
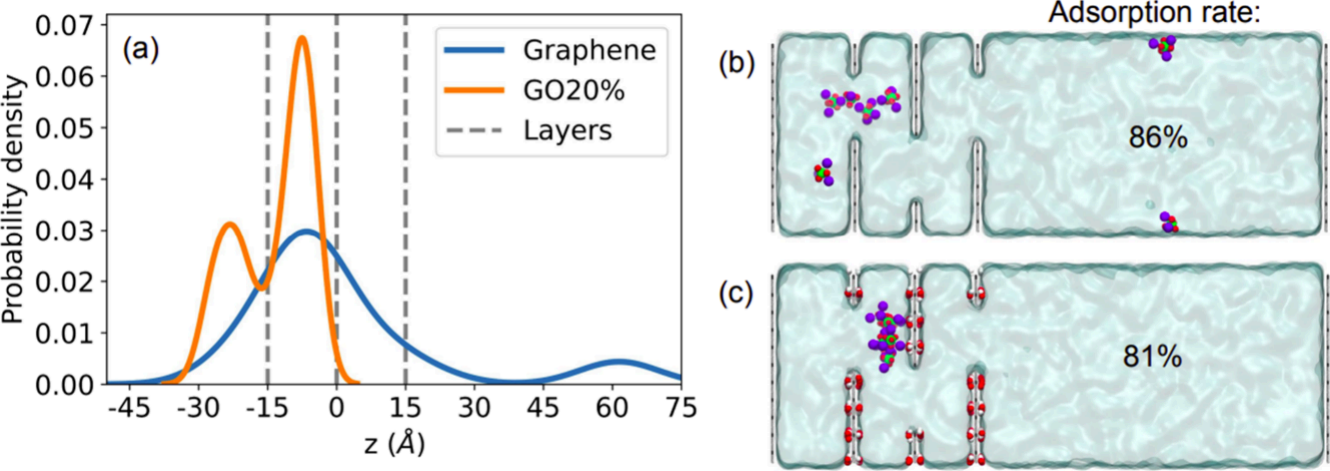
(a) Comparison between the phosphorus density profile of graphene and GO20%. Snapshots and adsorption rates for (b) Graphene and (c) GO20% are also shown.

**Figure 6:**
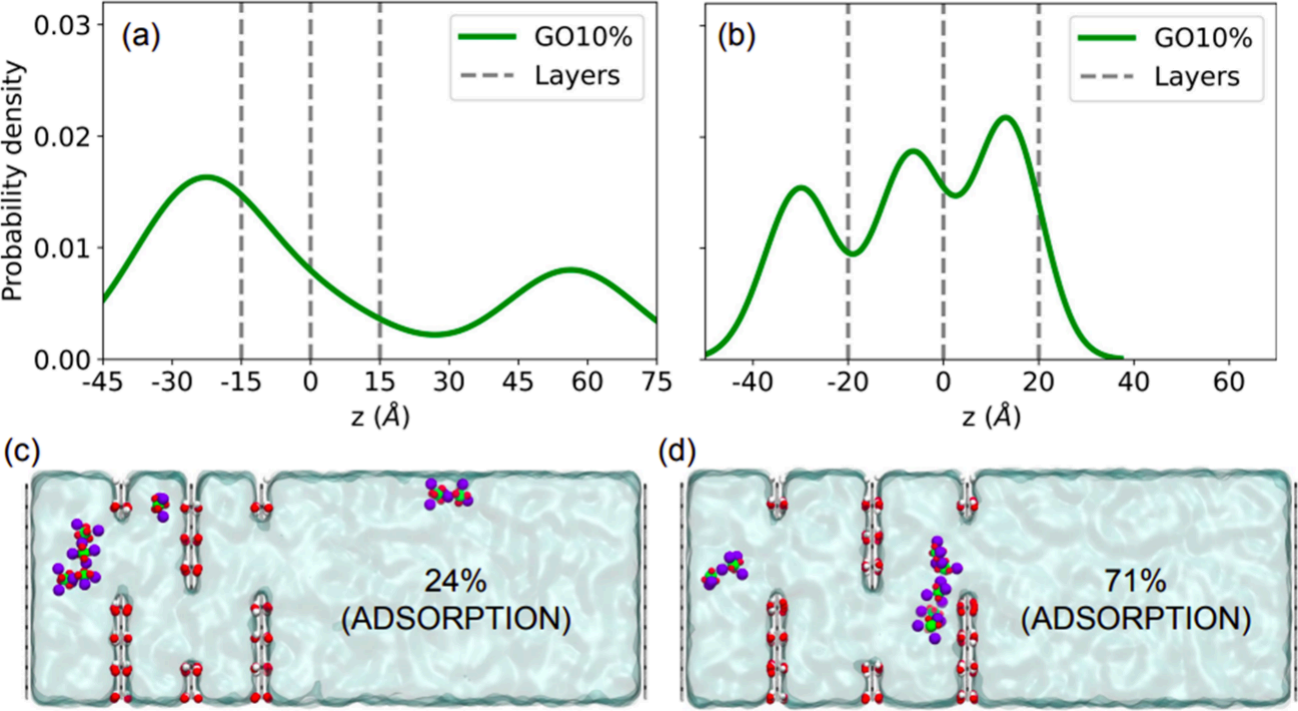
Phosphorus density profile along the GO10% simulation box with interlayer distances set to (a) 15 and (b) 20 Å, with snapshots of their respective final configurations in (c) and (d).

Figure 4 shows the radial distribution function (RDF) between K–P and P–P atoms extracted from the MD simulations. The formation of a potassium shell around phosphate molecules is observed in Figure 4a, strongly suggesting their aggregation. A second peak around 7 Å confirms the structuration of potassium in combination with phosphate. Following this process, we can observe in Figure 4b that phosphorus atoms also exhibit a first layer (indicated by the pronounced first peak at ∼5 Å) for all systems, confirming the clusterization of phosphate molecules in the aqueous solution. The phenomenon is very similar to the flocculation observed in ions in water^46^ and is found here to drive phosphate adsorption in the 3D-rGO membranes. The snapshots in Figures 5 and 6 represent the clusterization phenomena occurring in these systems.

Figure 5 shows the density profile along the simulation box (gray lines represent carbon positions) for graphene and GO20%. We can observe that they present similar adsorption of phosphate molecules (86 and 81%, respectively). However, it is important to note that GO20% systems do not let any phosphate molecule escape during the simulation. This is not the case for graphene, where we observe phosphate molecules passing through the membrane, as can be seen by the positive density profile after the membrane (*z* > 15 Å). Furthermore, all the ions are either adsorbed (already in between the first two layers) or rejected by the GO20% membrane, demonstrating the high efficiency of these systems. The result highlights the impact of graphene functionalization in membrane-mediated water purification processes.

Many experimental and theoretical contributions have shown that the interlayer distance is a determinant factor for the adsorption of ions and molecules in graphene-based membranes. Figure 6a,b show the density profile for GO10% with interlayer distances of 15 and 20 Å, respectively. We can see an increase in adsorption for larger distances, with very marked density peaks for an interlayer distance of 20 Å. As the interlayer positions are increased, there is more space for the phosphate molecules to form clusters; see the snapshots in Figure 6c,d. Again, clusterization is the main driving force of phosphate adsorption in these systems.

**Figure 7:**
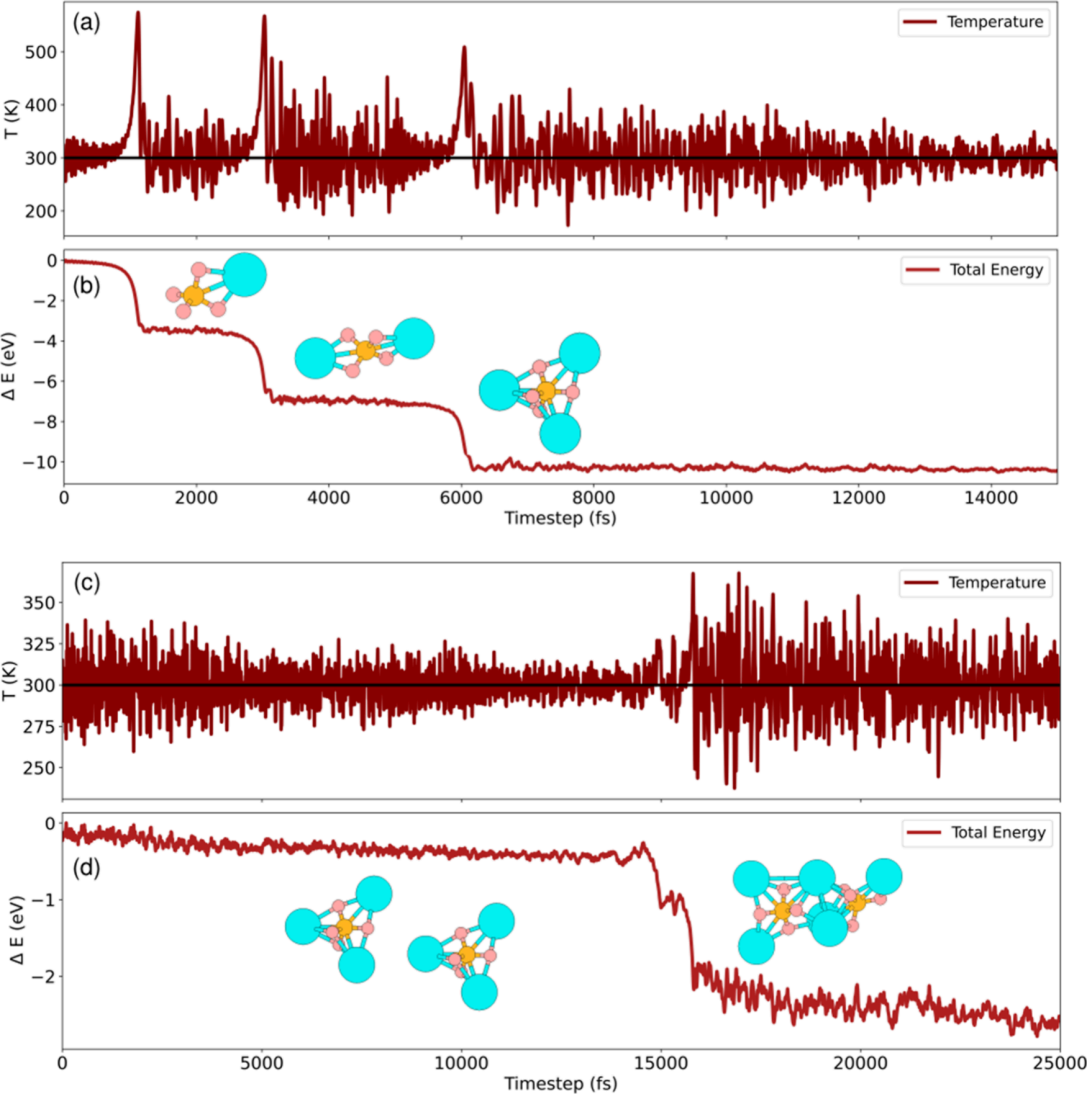
AIMD time evolution of (a) temperature and (b) total energy as K atoms (light blue spheres) are added to the phosphate structure (smaller orange and pink spheres). Time evolution of (c) temperature and, (d) total energy as two phosphate molecules are brought together.

In Figure 7, we show the results from AIMD simulations. We can observe a correlation between the peaks in temperature and the decrease in the total energy (Figure 7b) as more K atoms are bonded to the phosphate structure (see insets for instantaneous snapshots of the systems). This indicates that the ionic clusterization is energetically favorable, facilitating the increase of ionic clusters. It also shows the impact of temperature on the process, with consequences for the adsorption of phosphate, which is closely related to the experimental findings. We further simulated the intercluster interaction, as shown in Figure 7c,d. The results again show a temperature increase followed by a decrease in energy, highlighting thermodynamical impacts on the process where the aggregation of smaller phosphate clusters is energetically favorable. It is important to note that the clusterization process is believed to be the main driving mechanism behind ionic exclusion in nanoporous membranes.^35^ Additionally, we observed that once inside the membrane, the clusterization of phosphate molecules is enhanced for GO10% and GO20% (see Figure 4), which makes it harder for them to escape these membranes. These results corroborate the experimental findings that GO systems are more efficient in adsorbing phosphate than pristine graphene.

### Spectroscopic Studies

**Figure 8:**
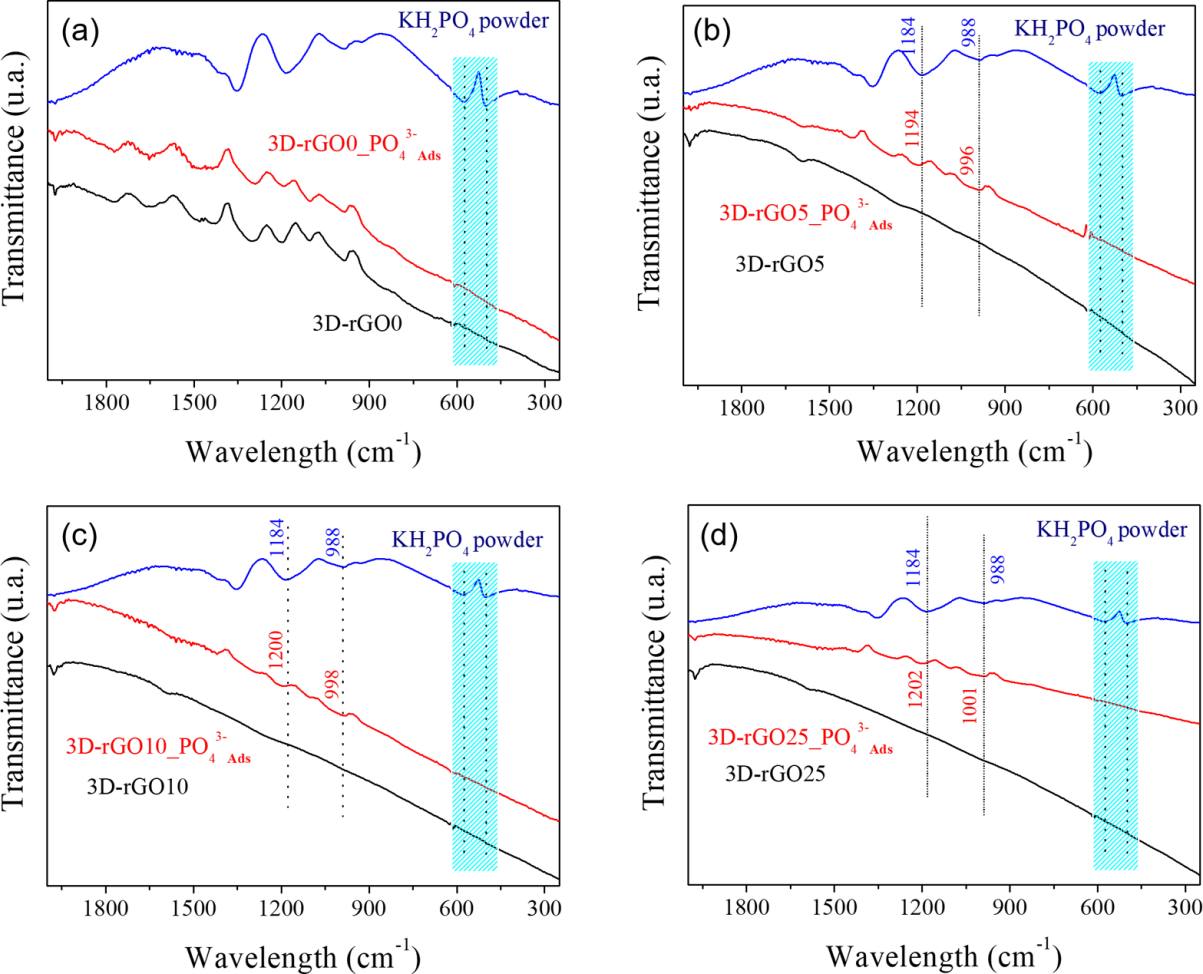
FTIR-ATR spectra before and after phosphate adsorption. (a) 3D-rGO0 (b) 3D-rGO5 (c) 3D-rGO10 and (d) 3D-rGO25.

The materials were evaluated by FTIR and Raman before and after adsorption to confirm the theoretical results. FTIR spectra are shown in Figure 8. It is possible to note the presence of several bands attributed to phosphate,^58−60^ confirming the reminiscence of the ions adsorbed inside the 3D-rGO porous structure.

An important observation for all the 3D-rGO materials after the adsorption is the disappearance of the bands associated with the out-of-plane POH bending modes between 600 and 500 cm^–1^.^60^ Additionally, in the spectra of 3D-rGO5, 3D-rG10, and 3D-rGO25 samples, a blue-shifting (up to 10 cm^–1^) of two strong phosphate bands, ca. 988 and 1184 cm^–1^ bands attributed to the O–P–O symmetric and asymmetric stretches, respectively. These FTIR observations indicate that the -POH bond no longer exists in the system and the O–P bond weakens; this probably can be due to a steric restriction by the formation of clusters and/or due to the adsorption of the phosphate ion occurs in a preferential conformation where the oxygen atoms are facing the graphene sheet, confirming the theoretical data.

**Figure 9:**
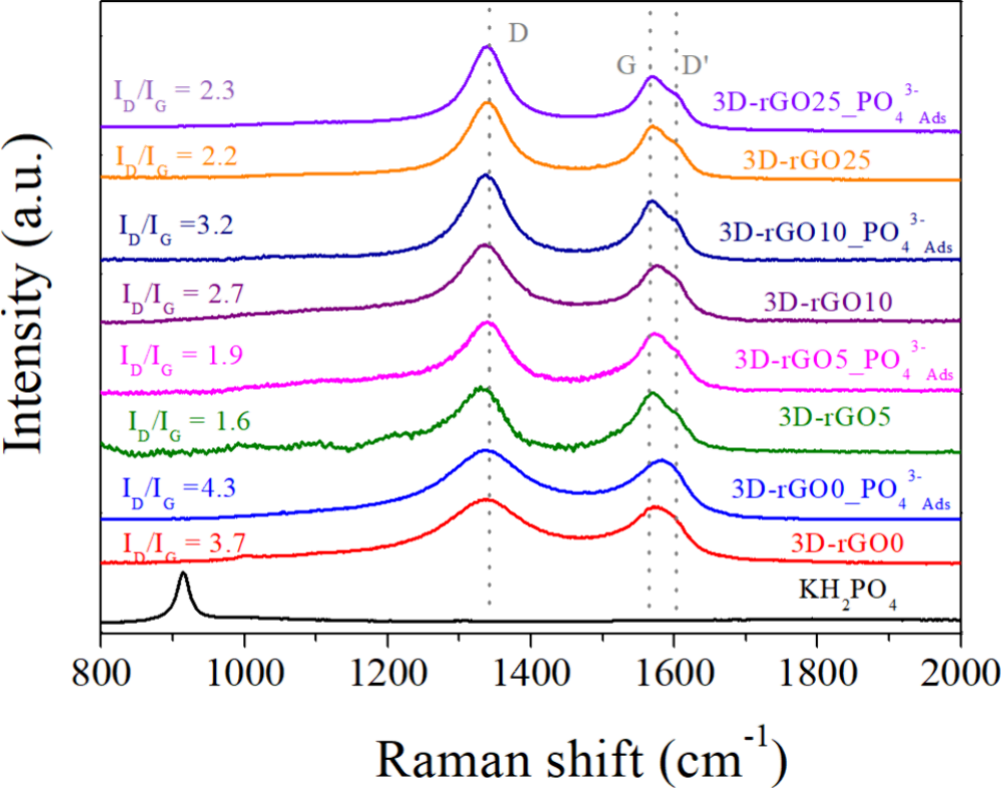
Raman spectra before and after phosphate adsorption for 3D-rGO0, 3D-rGO5, 3D-rGO10 and 3D-rGO25.

The Raman spectra are shown in Figure 9. After the adsorption for all the 3D-rGO, the D (1335 cm^–1^) and D′ band (1600 cm^–1^) become more prominent. These bands are associated with structural defects in the material, such as vacancies and functional groups. In this case, the phosphate bound to the material acts as a structural defect and intensifies the D and D′ band. The increase in *I*_D_/*I*_G_ ratios (Figure 9) after adsorption as a function of the oxidation degree confirms this observation. This ratio allows for estimating the number of structural defects present in the structure, indicating also a possible chemisorption mechanism in the present study. Furthermore, as shown in other works,^59^ in the case of experiments carried out at pH > pH_ZCP_, H_2_PO_4_^–^ and HPO_4_^–2^ are predominant in solution, decreasing the contribution of electrostatic attractions and intensifying the contribution of ligand exchange.

### Adsorption Mechanism

We have shown a lower efficiency of the more reduced material (3D-rGO25) and a trend toward better results with the more oxidized materials (3D-rGO0). The materials with intermediate reduction, 3D-rGO5 and 3D-rGO10, also show intermediate results. In addition, we observed a lower influence in the results of the 3D-rGO5 material for the variation of the temperature parameters.

Simultaneously analyzing the theoretical and experimental results, we verified an agreement between the results obtained. Theoretically, two main factors that influence the adsorption are the degree of oxidation of the structure and the pore sizes. The first factor corroborates the experimental data, showing that the more oxidized the structure, the better the adsorption results. Thus, theoretically and experimentally, the adsorptive capacity of materials follows the order 3D-rGO0 > 3D-rGO5 > 3D-rGO10 > 3D-rGO25.

As for the influence of the structure/pore size compaction, we have a greater compaction structure with the increase of the amount of reducing agent, so that, also, the adsorptive capacity of the materials follows the order 3D-rGO0 > 3D-rGO5 > 3D-rGO10 > 3D-rGO25. We found that the influence of pore size is linked to the clustering of phosphate molecules in the aqueous medium, which occurs due to their energetic viability. This clustering reduces the process of molecules entering the pores, limiting adsorption.

An important point to be considered concerns the adsorptive efficiencies of 3D-rGO0 and 3D-rGO5 materials. Previous works verified that the 3D-rGO5 material has better adsorptive capacities,^29,46−48^ which is not verified in this study. One explanation refers to the type of analyte investigated. While cationic dyes were better adsorbed on 3D-rGO5, this material presents an intermediate behavior. This is probably due to the lesser influence of oxygenated functional groups. The 3D-rGO0 material stands out in this work, presenting the largest and most significant amount of functional groups (Table 2). Despite having the advantage of a high degree of oxidation, this material has the disadvantage of not presenting a cohesive structure with a well-defined three-dimensional porous network. Thus, we verified that the influence of pore size is more significant for cationic dyes, and the presence of oxygen groups is fundamental for the adsorption of phosphate ions.

**Figure 10:**
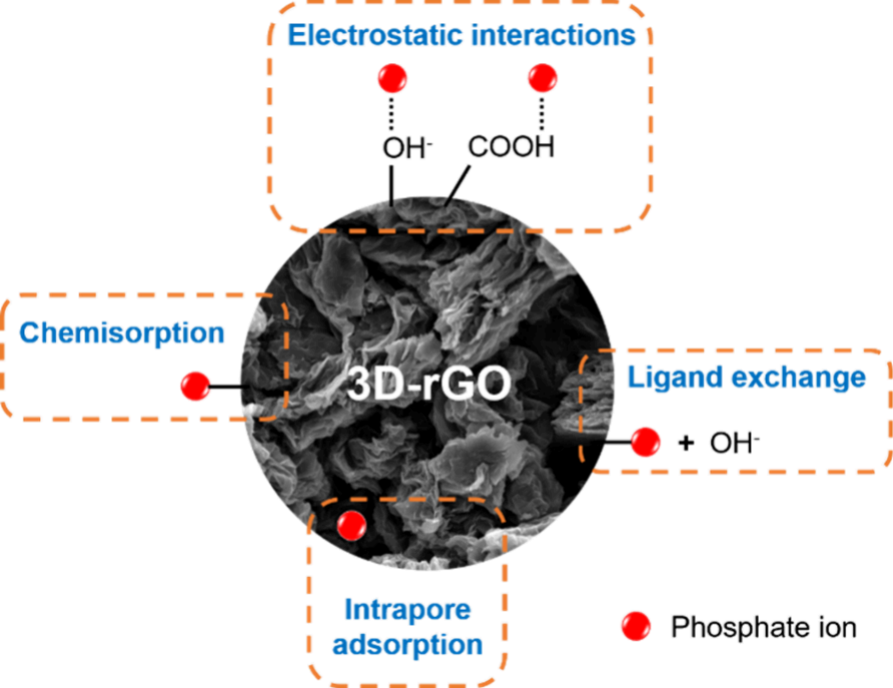
Possible phosphate ion adsorption mechanism in three-dimensional graphene-based materials.

Additionally, the theoretical results confirmed the exothermic nature of the adsorption process, adding to the experimental data to show the impact of temperature as an important parameter in the adsorption of the analyzed molecules. The calculations confirm the need for lower temperatures for the adsorption process to be more efficient. Finally, the pH variation results indicate that the adsorption mechanism could be considered a chemical process, as noted in the pH_ZCP_ of the four materials. In Figure 10, we suggest a schematic representation of the possible route for the adsorption mechanism in the systems studied here.

## Conclusions

For the experimental results, we observed that optimizing the pH, ionic strength, and temperature is essential to better removing phosphate ions from aqueous media. We observed an enhanced overall performance when the pH was neutral to basic, at low temperatures, and with NaCl. Furthermore, it was possible to evaluate those materials obtained with small synthetic variations that may present discrepant results according to the properties of each material. Thus, by combining experimental and theoretical results, we show that (i) the oxidation of graphene makes it more efficient in removing phosphate from water; (ii) both classical and quantum calculations (MD and AIMD simulations) show that clusterization of phosphate ions and molecules is energetically favorable, preventing its passage and reducing adsorption in materials with smaller pores (also allowing for the 3D-rGO0 material to adsorb more than 3D-rGO25); (iii) simulations, FTIR and Raman show that clusterization is the driving mechanism behind the adsorption performance of 3D-rGO; (iv) classical simulations confirm that larger interlayer distances in rGO lead to higher adsorption rates, and facilitated clusterization is again the reason behind it.

## Data Availability

All computational data is available under reasonable request.

## Supporting Information Available

More details about XRD and TGA characterizations, phosphate analytical curve and stability assessment (PDF)
