# Supplementary material for: Unraveling the Phosphorus Adsorption Mechanisms in Three-dimensional Reduced Graphene Oxide Materials
Source: Langmuir. 2024 May 16;40(21):11173–83. doi: 10.1021/acs.langmuir.4c00810 (PMC11140737; doi:10.1021/acs.langmuir.4c00810)
Supplement: Supplementary file 1 — la4c00810_si_001.pdf [file la4c00810_si_001.pdf]

Supporting Information

Unraveling the phosphorus adsorption mechanisms in three-dimensional reduced graphene oxide materials

Patrick R.B. Côrtes[a], Mayara Bitencourt Leão[b], Gabriel Lopes Rezende Reis[b], Douglas Duarte de Vargas[a], Gabriel Fidencio Murillo[c], Mateus Henrique Köhler[a], Carolina Ferreira de Matos Jauris[c*]

[a]*Department of Physics, Federal University of Santa Maria, Santa Maria, 97105-900, Brazil*

[b]*Environmental Science and Technology Center, Federal University of Pampa, Caçapava do Sul, 96570000, Brazil*

[c]*Department of Chemistry, Federal University of Santa Maria, Santa Maria, 97105-900, Brazil*

*CORRESPONDING AUTHOR:

Carolina Ferreira de Matos Jauris

Universidade Federal de Santa Maria

97105340– Santa Maria- RS - Brazil

Phone: +55- 55 3220-8000

E-mail: carolina.matos@ufsm.br

Number of pages: 4

Number of figures: 4

Number of schemes: 0

Number of tables: 1

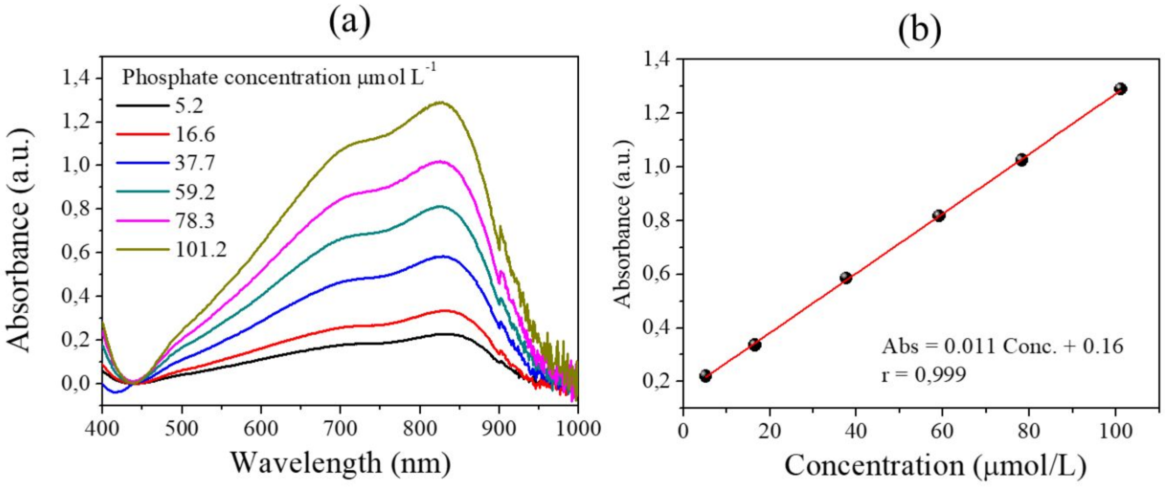

Figure S1. (a) Spectra obtained for the phosphate ion and curve (b) construction

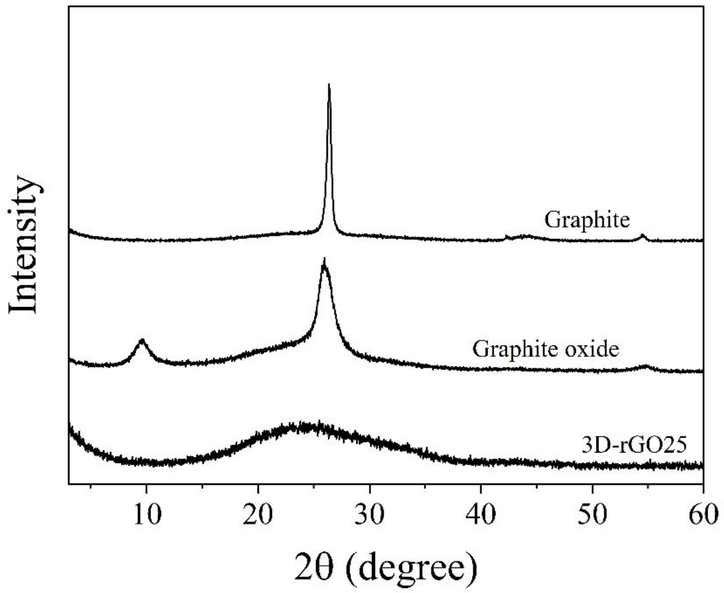

Figure S2. X-ray diffractograms of graphite, graphite oxide, and 3D-rGO25.

X-ray diffractograms of graphite, graphite oxide (Gr-O), 2D-rGO, 3D-rGO0, and 3D-rGO25 are shown in Figure S2. The peaks associated with the characteristic graphite planes are observed at 2θ = 26.38°, referring to the [002] plane, which corresponds to a distance of 0.34 nm between the graphene sheets in the structure, and at 2θ = 54.56 °, which refers to the [004] plan. For Gr-O, there is a peak at 2θ = 9.68°, with an interplanar distance of 0.92 nm, indicating an increase in the distance from the sheet after the oxidation process. However, the oxidation process was incomplete,

as the peak at 2θ = 24.4° (with the graphene sheets separated) could also be observed. After reducing the graphene to a three-dimensional format without adding a reducing agent (3D-rGO0), we observed a discrete and very wide diffraction peak at 2θ = 24°, which indicates a considerable variation in the interplanar distances.

Furthermore, a peak at 2θ = 9.68° indicates a distance resulting from the presence of oxygenated functional groups. The diffractogram of this material is very similar to that of Gr-O, indicating that the thermal reduction process is not as efficient as the thermochemical process, and the distance between the sheets is still significant. For the 3D-rGO25 material, a broad diffraction peak centered at 2θ = 23.8° is found, indicating that the graphene sheets are rearranged but not stacked in the three-dimensional structure. Thus, 3D materials have various interplanar distances and a different arrangement from the original graphite due to oxidation of graphite, exfoliation of graphite oxide to graphene oxide, and reduction to reduced three-dimensional graphene oxide, confirming the results observed by SEM.

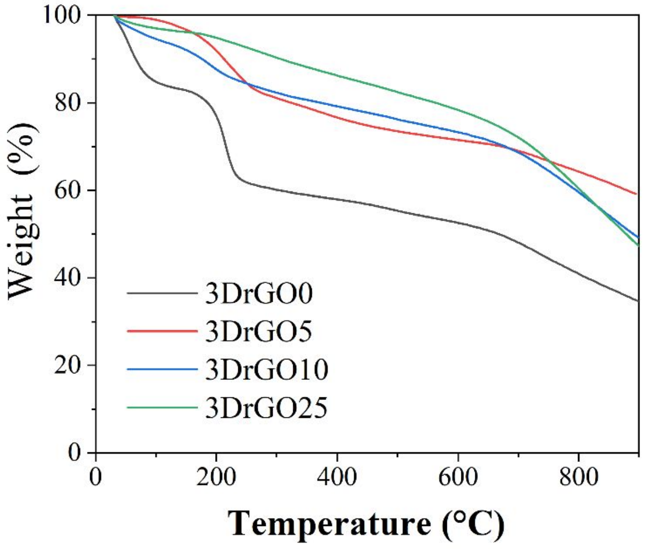

Figure S3. TGA curve of 3D-rGO0; 3D-rGO5; 3D-rGO10 and 3D-rGO25 collected in nitrogen atmosphere.

Thermogravimetric analyses (TGA) were carried out on a Netzsch TG 209 F1 equipment under a nitrogen atmosphere, starting from room temperature up to 900 ºC, at a heating rate of 10 ºC min^{-1}. Significant differences were observed in the thermal properties of the materials. When no reducing agent was added, two mass loss events were observed. The first, relating to the water loss, starts from room temperature near to 100 °C, and the second up to ~300 °C, corresponding to eliminating oxygenated functional groups. Adding a reducing agent makes the mass loss up to 200°C less significant, resulting in a more thermally stable structure. For all materials, a third mass loss event is observed associated with the oxidation of the graphite structure, between 300 and 800 ºC. The mass loss event associated with the amount of oxygenated groups in the materials is presented in detail in Table S1, where the greater thermal stability of smaller samples is seen.

Table S1. Mass loss events for the four materials.

Material	100-300°C (wt% loss of oxygenated groups)
**3D-rGO0**	26.1
**3D-rGO5**	11.4
**3D-rGO10**	8.5
**3D-rGO25**	5.1

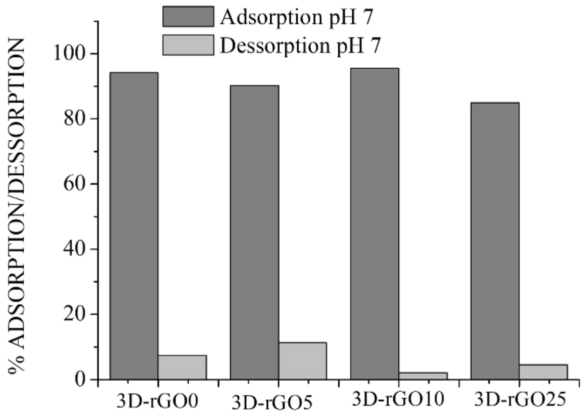

Figure S4. Stability assessment by comparing the percentages of phosphate ions adsorbed and desorbed after 24 hours at pH 7.
